# Isolation and characterization of an astrovirus causing fatal visceral gout in domestic goslings

**DOI:** 10.1038/s41426-018-0074-5

**Published:** 2018-04-19

**Authors:** Qingshui Zhang, Yanxin Cao, Jun Wang, Guanghua Fu, Mengxu Sun, Lijiao Zhang, Li Meng, Guolin Cui, Yu Huang, Xueying Hu, Jingliang Su

**Affiliations:** 1 Key Laboratory of Animal Epidemiology and Zoonosis, Ministry of Agriculture, College of Veterinary Medicine, China Agricultural University, Beijing 100193, China; 2 Institute of Animal Husbandry and Veterinary Medicine, Fujian Academy of Agricultural Sciences, Fuzhou 350013, Fujian China; 3 College of Veterinary Medicine, Huazhong Agricultural University, Wuhan 430070, Hubei China

## Abstract

Astroviruses are recognized as a leading cause of gastroenteritis in humans and animals. They are also associated with extra-intestinal diseases, such as hepatitis in ducklings, nephritis in chickens, and encephalitis in cattle. In February 2017, a fatal infection of goslings characterized by visceral urate deposition was reported in the Shandong province, China. Our systematic investigation led to the isolation of an astrovirus, designated AAstV/Goose/CHN/2017/SD01, and similar disease was reproduced by experimental infection of healthy goslings, fulfilling Koch’s postulates. The isolated astrovirus replicated well and resulted in 100% mortality of goose embryos. Complete genome sequence analysis revealed that the isolate was genetically distinct from known astroviruses and closely related to members of the avastrovirus genogroup II. Experimental infection showed that the isolate was highly pathogenic in goslings, causing clinical signs, growth repression and in many cases mortality. Histopathological examination indicated that lesions occurred mainly in the kidneys of infected birds. However, virus-specific genomic RNA was detected in all representative tissues, and virus shedding was detected up to 12 days after inoculation, suggesting that the isolate was able to spread systemically and replicate efficiently in vivo. Collectively, our study demonstrates, for the first time, the etiological role of a genetically distinct astrovirus in the fatal infection of goslings.

## Introduction

Astroviruses (AstVs) are non-enveloped, positive-sense, single-stranded RNA viruses belonging to the *Astroviridae* family. Currently, two genera: namely *Mamastrovirus* and *Avastrovirus* are distinguished within this family. The genus *Mamastrovirus* includes astrovirus species isolated from humans and a number of mammals. Isolates originated from avian species, such as turkey, chickens, ducks, and other birds are classified into the genus *Avastrovirus*^1, 2^. AstVs have been detected in humans and a variety of animal species, including non-human primates, other mammals and avian species^3–5^. Their genomes are 6.8–7.9 kb in length, consisting of a 5′-untranslated region (UTR), three open reading frames (ORFs), a 3′-UTR and a poly (A) tail^6^. The high degree of genetic diversity among AstVs and their recombination potential signify their capacity to cause a broad spectrum of diseases in multiple host species^3, 7, 8^. Human classical AstVs are a frequent cause of acute gastroenteritis in young children and the elderly, occasionally with encephalitis^8^.

In poultry, AstV infections have been found to be associated with multiple diseases, such as poult enteritis mortality syndrome, runting-stunting syndrome of broilers, white chick syndrome, kidney and visceral gout in broilers and fatal hepatitis of ducklings, leading to substantial economic losses^9–16^. Increasing evidence indicates that there is a high degree of cross species transmission of AstVs between domestic birds, and even the potential to infect humans^17^. By comparison, fewer AstV infection cases have been described in domestic goose flocks. Bidin et al.^18^ reported the detection of avian nephritis virus infection in Croatian goose flocks and provided evidence that this AstV was associated with stunting and pre-hatching mortality of goose embryos. Studies to detect AstV genomes from the clinical samples of geese suggested that these viruses might distribute widely among goose flocks, as seen in other poultry flocks^19, 20^. In February 2017, an outbreak of disease was reported in a goose farm in Weifang, Shandong Province, China. Affected flocks (containing 2000–3000 goslings) experienced continuous mortality rates ranging from 20 to 30% during the first 2 weeks of the outbreak despite antibiotic and supportive treatment. We conducted a systematic investigation to identify the causative agent of this disease and report here the isolation and characterization of a genetically distinct avian AstV. The pathogenicity of this virus was evaluated by experimental infection of goslings.

## Results

### Case history and microbiological examination of the field samples

**Fig. 1:**
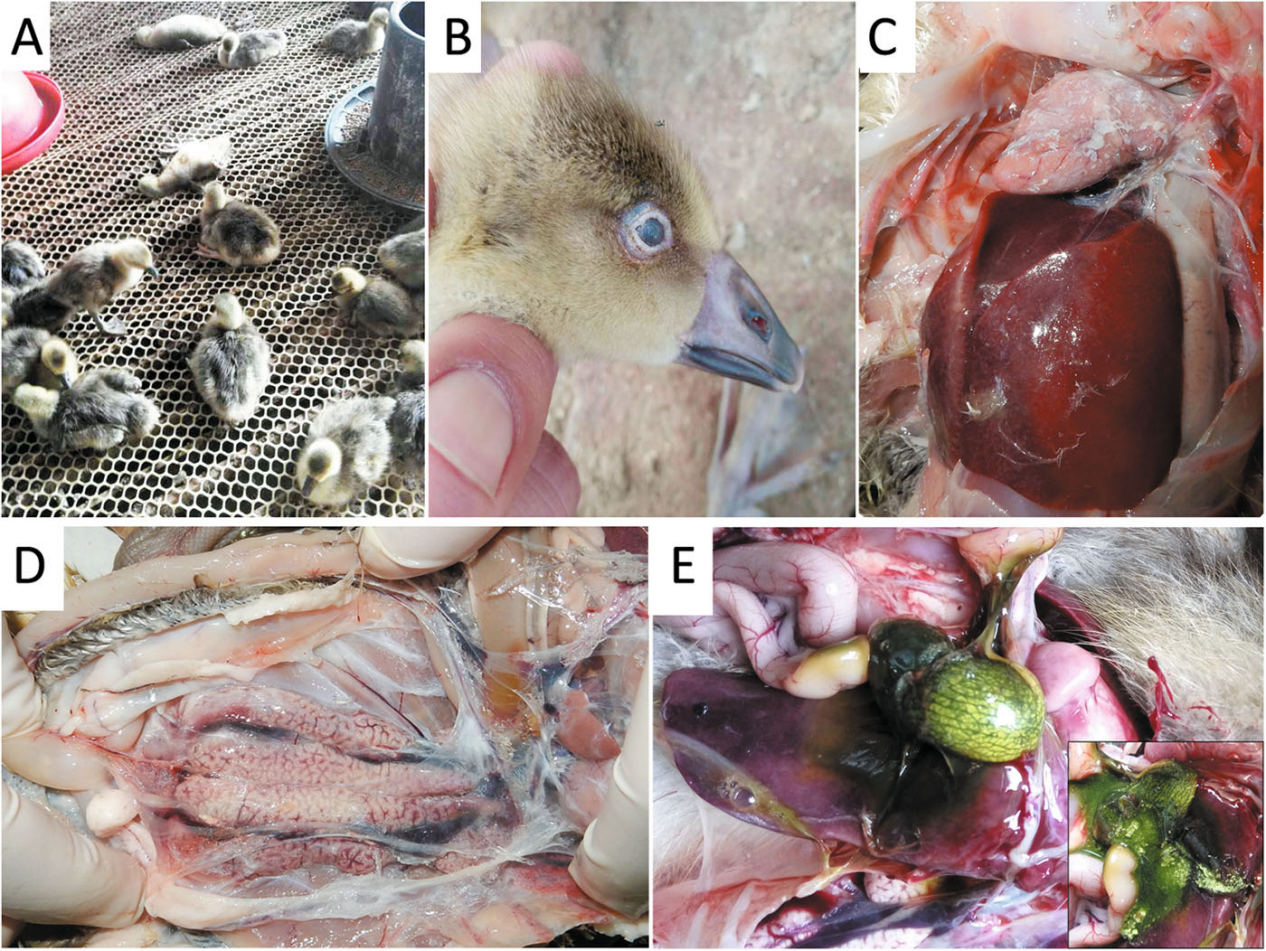
Clinical signs and postmortem lesions of goslings infected in the field. **a** Goslings appear depressed and lethargic. **b** Infected goslings with gray-white cloudy palpebra tertia. **c** Visceral urate deposition over the heart and liver. **d** Urate deposition and swollen kidney. **e** Distended bile sac and urate particles (insert)

In the field, affected goslings displayed signs of depression and were observed sitting alone (Fig. 1a). The palpebra tertia of some of the goslings showed an obvious gray-white cloudy appearance (Fig. 1b). Death occurred from when the goslings were 5–6 days old, and peaked at 12–13 days old; then the mortality rate decreased gradually to the end of the third week. A common feature at postmortem was visceral urate deposition on the serous surfaces of the heart, liver and kidney (Fig. 1c, d). Distended bile sacs with abundant urate particles were also observed (Fig. 1e).

**Fig. 2:**
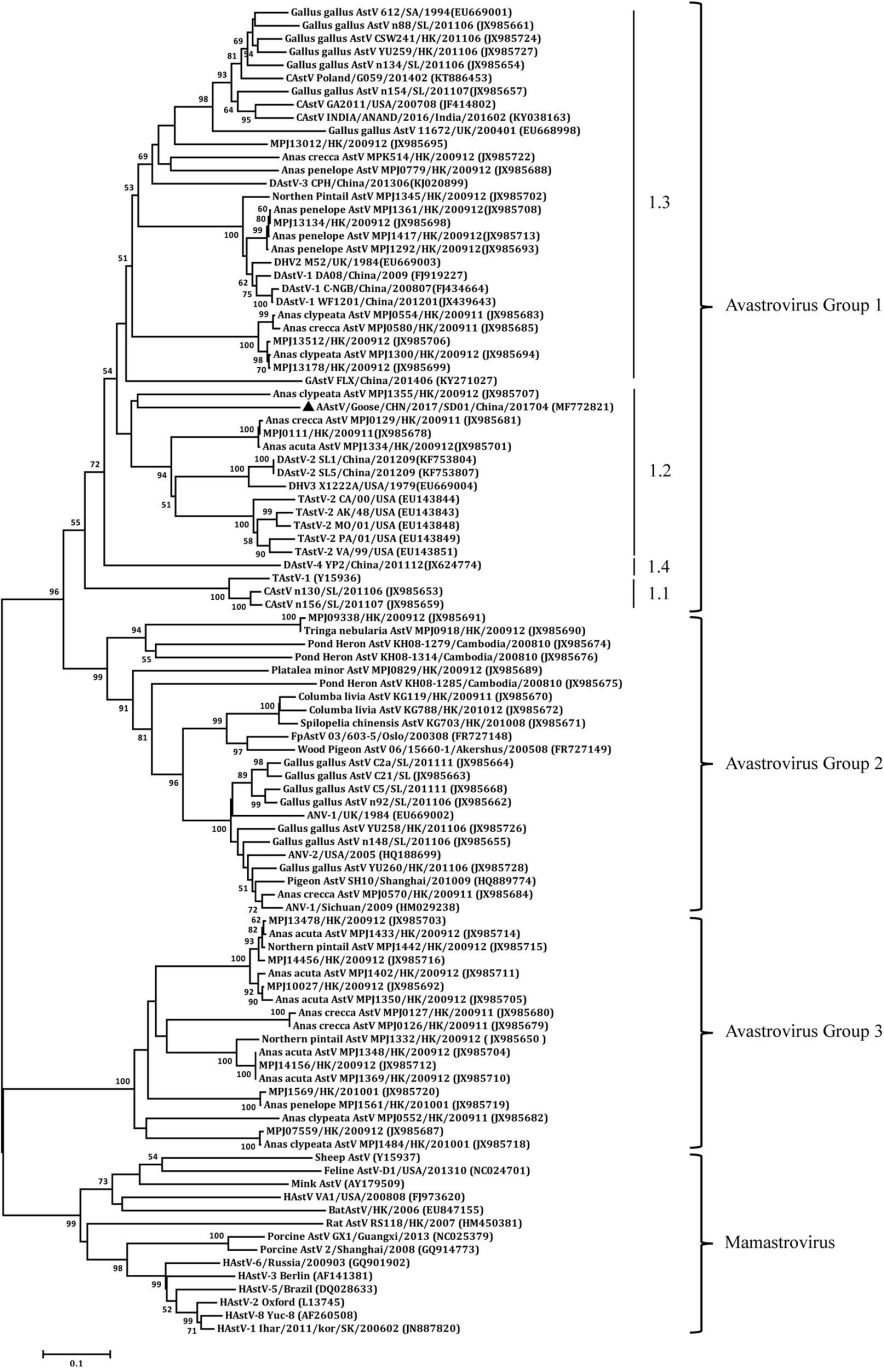
Phylogenetic analysis on *RdRp* genes of astroviruses using MEGA 7.0. The tree was constructed based on about 391 nt (nucleotide) sequence, by using the Neighbor-joining method with 1000 bootstrap replicates and Maximum Composite Likelihood model. Node labels indicate bootstrap values, and bootstrap values <50% were hided. The location (HK Hong Kong, SL Sri Lanka, UK United Kingdom, SK South Korea, SA South Korea) and time of sample collected was shown. The AAstV/Goose/CHN/2017/SD01 isolate determined in this work is indicated by a black triangle

**Fig. 3:**
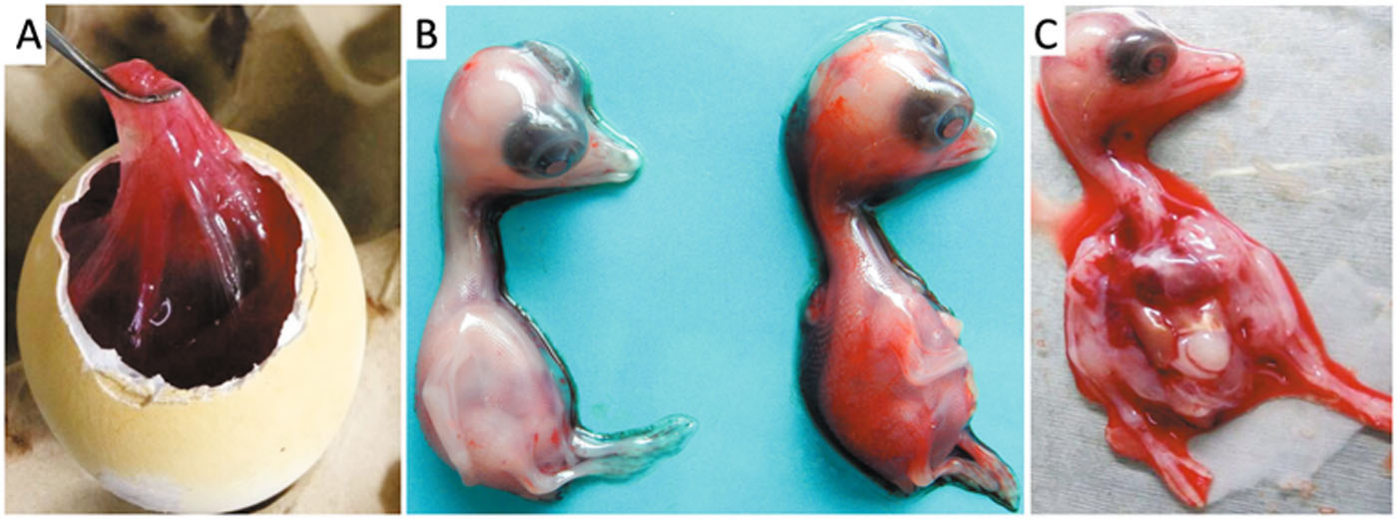
Gross lesions of goose embryos infected with goose astrovirus. **a** Edema of the chorioallantoic membrane. **b** Uninfected embryo (left) and infected embryo with subcutaneous hemorrhage (right). **c** Infected embryo with liver necrosis

Virulent bacteria were not isolated and tissue samples were negative by PCR for goose parvovirus, goose hemorrhagic polyomavirus, reovirus, or Tembusu virus. However, a DNA fragment was amplified from the RNA sample extracted from the pooled spleen, liver and kidney tissues using pan-AstV RT-PCR targeting the AstV RNA-dependent RNA polymerase (*RdRp*) gene^21^. Sequence and phylogenetic analysis of the amplified *RdRp* gene with other known AstV sequences retrieved in the GenBank database showed that the detected virus could be assigned to the subgroup 1.2 within the avastrovius group 1, with the closest relationship to the astrovirus detected from dropping samples of northern shovelers (*Anas clypeata*) in Hong Kong (Fig. 2)^22^. However, the nucleotide sequence of the *RdRp* gene had ≤67.5% similarity to the sequences of other astroviruses within avastrovirus group 1, suggesting that the virus was genetically distinct from known avastroviruses. Therefore, the isolation of AstV was initiated by inoculating tissue samples into goose embryos. For the first inoculation, significant thickening of the embryo’s chorioallantoic membrane was noted although no death occurred by 5 days post inoculation (dpi). The subsequent passage of the isolate caused 60–100% mortality of the embryos by 5 dpi. The dead embryos exhibited severe subcutaneous edema and hemorrhages with necrotic foci in the liver (Fig. 3). Using the gene specific RT-PCR, the AstV was consistently detected in the allantoic fluids. Quantal assays showed that the infectious virus titers of the embryo allantoic fluid increased from 5 × 10^4^ ELD_50_/ml for the fourth passage to 5 × 10^5.5^ ELD_50_/ml for the 9^th^ passage, indicating that the isolates adapted to the goose embryo culture system. Therefore, the isolate was designated AAstV/Goose/CHN/2017/SD01 (SD01 hereafter) as proposed by Martella et al.^23^.

### Genome sequence analysis

**Fig. 4:**
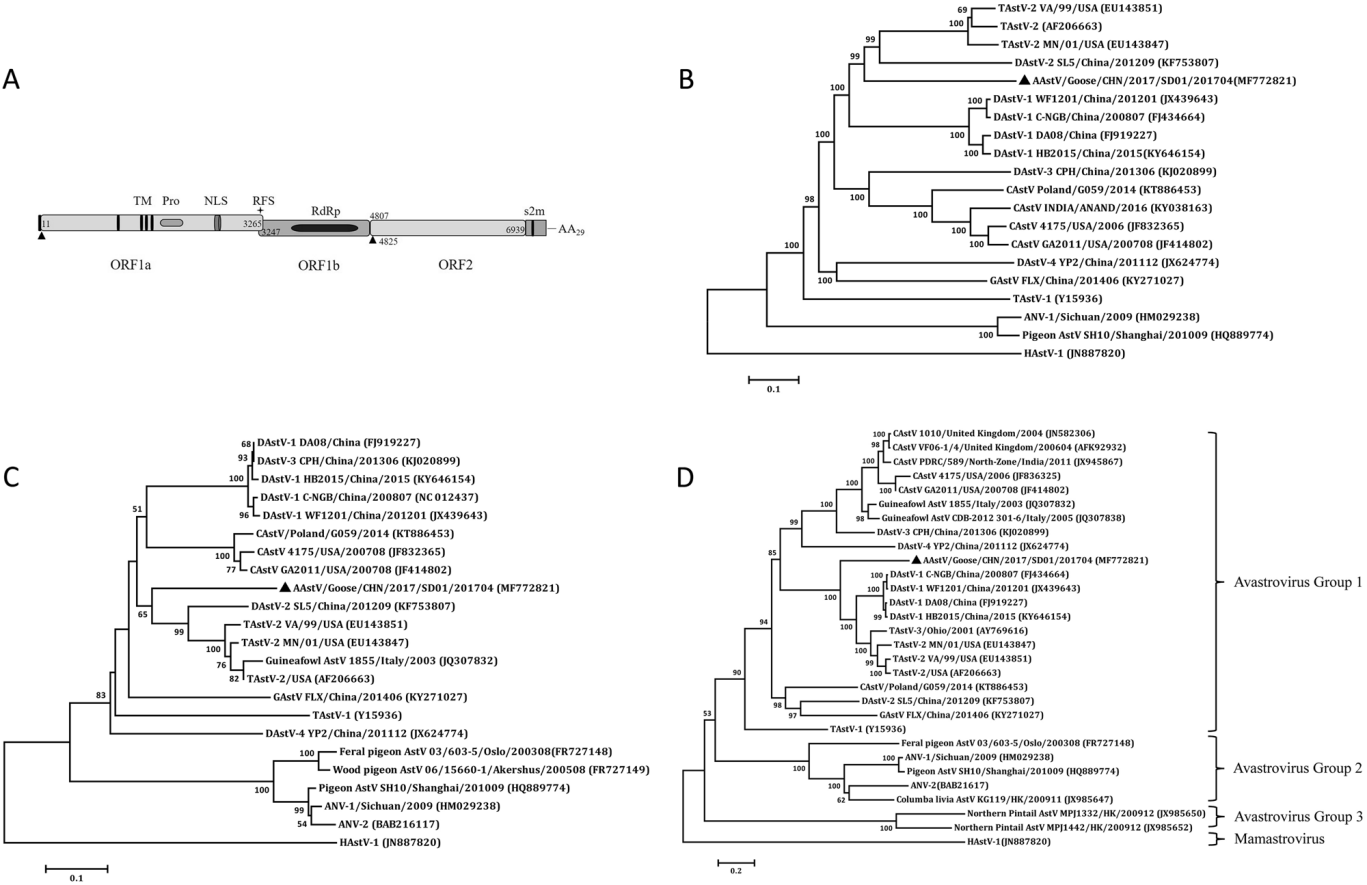
Genomic and phylogenetic analysis of goose astrovirus. **a** Predicted genome organization of goose astrovirus AAstV/Goose/CHN/2017/SD01. The translation start sites of two ORFs are indicated by black triangles. **b** Phylogenetic relationship analysis based on the nucleotide sequences of the complete genome. **c** Phylogenetic relationship analysis based on the amino acid sequences of ORF1b and ORF2 (**d**). Human astrovirus (HAstV-1) was used as an outgroup. The trees were generated using MEGA 7.0 software and the Neighbor-joining method with 1000 bootstrap replicates. GenBank accession numbers of the sequences are indicated in parentheses. The AAstV/Goose/CHN/2017/SD01 isolate determined in this work is indicated by a black triangle

The complete genome of the SD01 was identified by sequencing of the RT-PCR products and was submitted to the GenBank database under accession number MF772821. The genome was 7175 nucleotides (nt) in length with similar gene organization to other known avastroviruses, consisting of a 5′-UTR of 10 nt, three sequential ORFs (ORF1a, ORF1b and ORF2), a 3′-UTR of 236 nt and a poly (A) tail stretching 30 nt (Fig. 4a). ORF1a of the isolate was 3255 nt long, encoding a polypeptide of 1084 amino acids (aa) with 27.9–59.5% identity to corresponding regions of other known avian AstVs as determined by BLAST analysis (Table 1). The predicted nonstructural protein contained a trypsin-like peptidase domain as revealed by Pfam analysis with a serine protease motif at position 672 (GNSG), a nuclear localization signal motif at position 773 (KKKGKTK), and four predicted transmembrane domains. As is the case with other known avian AstVs, there was an overlapping region between ORF1a and ORF1b (3247–3265 nt), which contains the highly conserved ribosome frameshift sequence (5′-AAAAAAC-3′) and a downstream hairpin structure (3270–3295 nt) as predicted by RNA folding analysis. ORF1b was 1560 nt long and was predicted to encode a RNA-dependent RNA polymerase. There was an 18 nt spacer between the stop codon of ORF1b and the start codon of ORF2. ORF2 was 2133 nt long encoding a capsid protein of 704 aa. A stem-loop-II-like (s2m) motif consisting of 43 nt was revealed adjacent to 10 nt of ORF2 in the 3′-UTR by Rfam analysis.

**Table 1:** Comparison of the nucleotide and amino acid identities of AAstV/Goose/CHN/2017/SD01 with selected representative astroviruses

Astrovirus (GenBank accession no.)	Percent identity(%) to AAstV/Goose/CHN/2017/SD01 (MF772821)			
	Genome (nt)	ORF1a (aa)	ORF1b (aa)	ORF2 (aa)
TAstV-2 VA/99/USA (EU143851)	62.4	59.5	68.7	57.6
TAstV-3/ Ohio/2001 (AY769616)	NA	NA	NA	56.4
DAstV-1 C-NGB/China/200807 (FJ919227)	60.0	48.6	64.3	56.2
GAstV FLX/China/201406 (KY271027)	58.1	47.7	61.0	42.5
TAstV-1 (Y15936)	54.6	40.6	56.0	41.3
DAstV-2 SL5/China/201209 (KF753807)	60.3	58.7	68.2	38.1
DAstV-3 CPH/China/201306 (KJ020899)	58.7	50.0	65.6	37.6
CAstV GA2011/USA/200708 (JF414802)	57.9	49.3	65.2	37.6
CAstV/Poland/G059/201402 (KT886453)	58.0	49.2	65.2	37.5
GfAstV CDB-2012 301-6/Italy/2003 (JQ307838)	NA	NA	NA	36.2
DAstV-4 YP2/China/201112 (JX624774)	56.7	42.6	61.3	34.2
ANV-2 /USA/2009 (HQ188699)	NA	NA	NA	31.8
Pond Heron AstV KH08-1279/HK/200810 (JX985649)	NA	NA	NA	31.1
Wood pigeon AstV 06/15660-1/Oslo/200308 (FR727147)	NA	NA	NA	28.3
ANV-1/Sichuan/2009 (HM029238)	51.5	28.6	52.3	28.5
Columba livia AstV KG119/HK/200911 (JX985647)	NA	NA	NA	28.2
Pigeon AstV SH10/Shanghai/201009 (HQ889774)	52.0	27.9	52.3	27.6
Northern pintail AstV MPJ1442/HK/200912 (JX985652)	NA	NA	NA	24.4
Northern pintail AstV MPJ1332/HK/200912 (JX985650)	NA	NA	NA	23.2
HAstV (JN887820)	49.1	20.7	38.2	19.7

*NA* the complete ORF is not available, *HK* Hong Kong

To determine the potential genetic mutation(s) that might occur during the goose embryo passage, the initial virus genome was sequenced using the total RNA extracted from the clinical case tissue homogenate. Nucleotide differences between the initial virus genome and that of the fourth embryo-passaged isolate was shown in Table S1. A single-mutation exhibited in the ORF2 gene of the adapted isolate, leading to the amino acid change from R_225_ to Q_225_. The potential effect of the mutation on the virus adaptation need to be further evaluated.

### Similarity of the isolate with known avian AstVs

The complete genome sequence of AstV SD01 had the highest similarity to those of turkey AstV 2 (TAstV-2) strains, at the level of 61.6–62.4% (representative TAstV-2 VA/99 in Table 1). The next was the duck astrovirus-2 (DAstV-2) SL5, with 60.3% nucleotide identity. Phylogenetic analysis of the full-length sequences showed that the SD01 formed a sister clade neighboring DAstV-2 and TAstV-2 in the avastrovirus genogroup II (Fig. 4b). Further analysis with the complete amino acid sequence of RdRp (Fig. 4c) and the capsid protein (Fig. 4d) revealed close alignment and closely matched phylogenetic trees.

The pairwise comparison of nucleotide and amino acid identities of the three ORFs among the representative avastrovirus isolates was shown in Table 1. Based on the available complete sequences of avian AstV strains, the amino acid of ORF1a, ORF1b, and ORF2 of SD01 shared the highest identities of 59.0–59.9%, 68.3–68.7%, and 55.3–57.7%, respectively, with the TAstV-2 strains. The mean amino acid genetic distance (p-dist) values based on the analysis of complete capsid protein with the representative isolates TAstV-2, TAstV-3 and DAstV-1 were 0.423–0.435 (Table S2). According to the species demarcation criteria in the genus avastroviruses (p-dist range between genotypes range between 0.576 and 0.741), the SD01 was grouped within the genotype consisting of TAstV-2, TAstV-3, and DAstV-1^2^. However, the p-dist values between TAstV-2, TAstV-3, and DAstV-1 included in this genotype was much lower, ranging between 0.162 and 0.293 (Table S2). These results suggested that SD01 has a higher variability than those previously detected avastroviruses in the genotype.

### Outcome of infection experiments

#### Gosling infection experiment 1

**Fig. 5:**
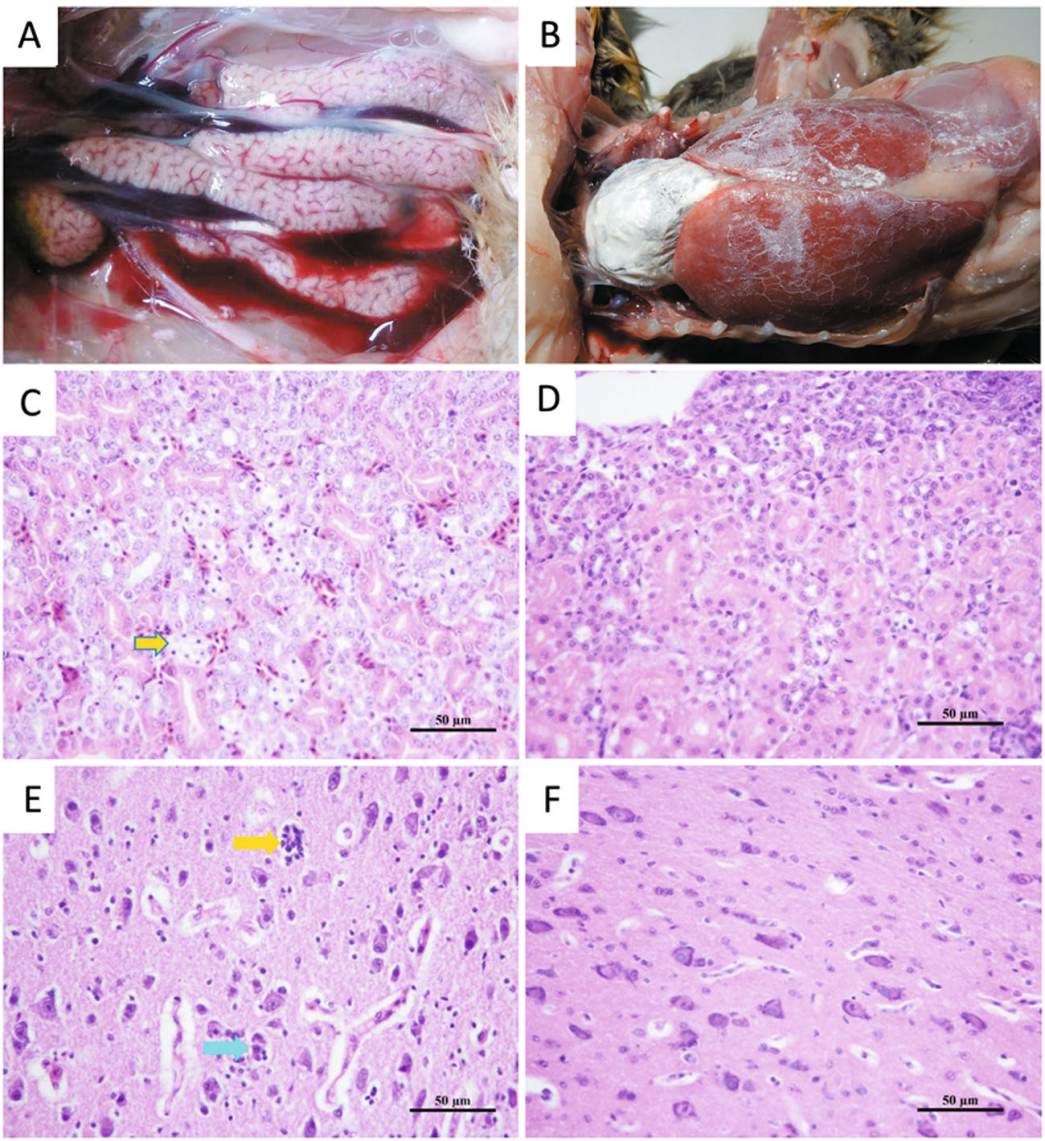
Postmortem lesions of goslings that died following experimental infection. **a** Mild swelling of kidneys observed in a gosling infected at 2 days old. **b** Urate deposition over the heart and liver of a dead gosling infected at 5 days old. **c** Renal tubular necrosis (yellow arrow) in a dead gosling infected at 2 days old (H&E). **d** Normal kidney section of an uninfected gosling (H&E). **e** Neuronophagia (blue arrow) and microgliosis (yellow arrow) in the cortex of the cerebrum and the dying neuron surrounded by satellite microglia in a gosling infected at 2 days old (**H**&**E**). **f** Normal brain section of an uninfected gosling (H&**E**)

Seven out of the 13 infected goslings displayed signs of depression from 3 to 8 dpi. One bird died at 4, 5, and 6 dpi, respectively, resulting in a mortality rate of 23% (3/13) during the experimental period. At necropsy, slight to moderately swollen kidneys were noted for the deceased birds (Fig. 5a). Histologic examination revealed degeneration and necrosis of the epithelial cells of the tubules of the kidneys (Fig. 5c). Neuronophagia and microgliosis was detected in the cortex of the cerebrum and the dying neuron was surrounded by satellite microglia (Fig. 5e). Following embryo inoculation, the inoculated virus was re-isolated from the liver and kidney tissues and confirmed by RT-PCR.

#### Gosling infection experiment 2

Infected goslings exhibited signs of depression from 3 dpi and this symptom persisted for 3–4 days. One bird died at 5 dpi and severe urate deposition, similar to that seen in the field cases, was evident on the surface of the heart, liver, and kidney (Fig. 5b). For the three infected goslings killed at 5 dpi, no evident gross lesion was noted in the visceral organs at postmortem. However, histologic examination revealed the presence of an eosinophilic proteinaceous substance in the renal tubules, and mild interstitial lymphocyte infiltration was noted in sample of two goslings (2/3) (Fig. S1). AstV RNA were detected in the collected tissues of three goslings (Fig. S2), indicating that the isolate has a wide tissue tropism after infection. All tissues from the uninfected birds were normal.

**Fig. 6:**
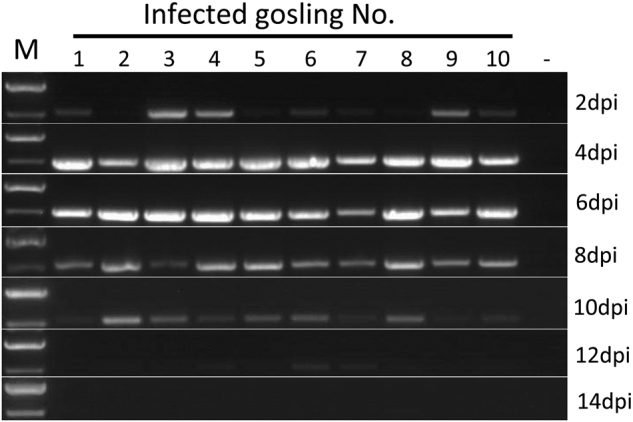
Photograph of the virus shedding pattern detected by RT-PCR of cloacal swabs from infected goslings. M DNA marker; dpi days post-infection

**Fig. 7:**
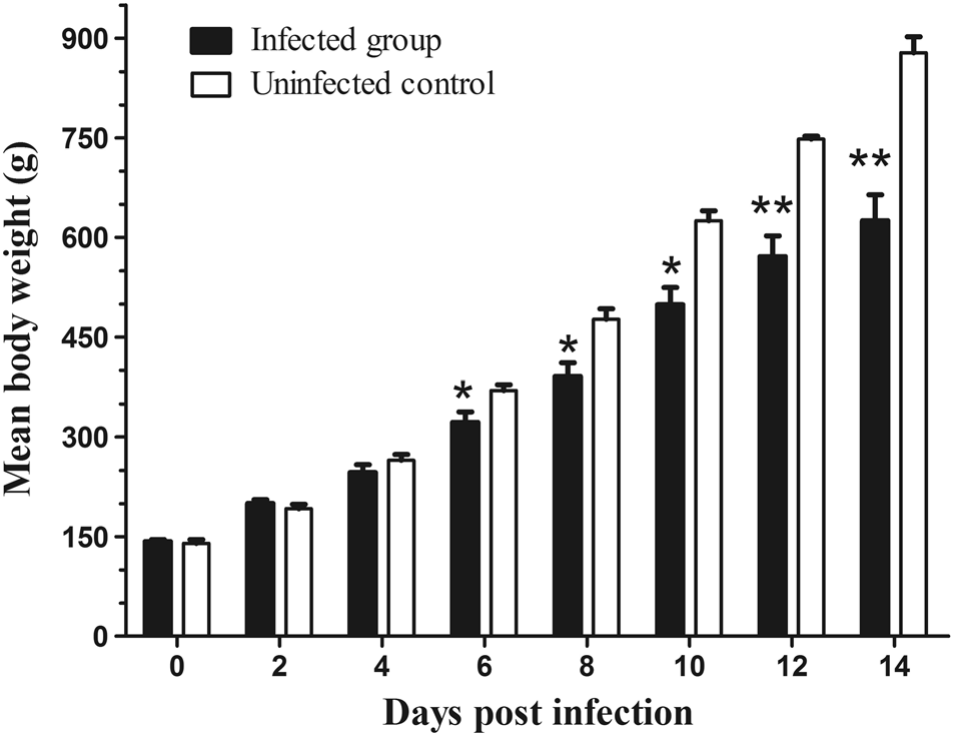
Histogram showing the average body weights of infected and control goslings. All values are presented as the mean body weight ± SD. *P*-values were calculated using Student’s *t*-test. *p < 0.05; **p < 0.01

When the samples were tested by RT-PCR for virus shedding evaluation, the AAstV specific RNA was sequentially detected from the cloacal swabs of infected goslings from 2 to 12 dpi (Fig. 6). Viral RNA could still be detected in the liver and spleen when the infected birds were killed at 15 dpi. Neither viral shedding nor positivity in the tissue samples was detected in the uninfected control goslings during the experiment. Infected goslings showed decreased body weight gain and the average body weight of infected birds was statistically significantly lower than that in the uninfected group from 6 dpi to the end of the experiment (Fig. 7). The average body weight in the infected group was 322 ± 73 g versus 370 ± 15 g in the control group at 6 dpi, and 625 ± 180 g versus 878 ± 48 g at 14 dpi, respectively.

#### Gosling infection experiment 3

Orally inoculation of goslings with the isolate resulted in depression of 4 birds from 5 to 8 dpi. One gosling died at 7 dpi with evident urate deposition on the surfaces of heart and liver at necropsy. Another severely ill bird was killed humanely at 8 dpi for animal welfare reasons. Viral RNA was detected by RT-PCR in the cloacal swabs from 4 dpi (Fig. S3) and growth depression was noticed in this group (Fig. S5). For the goslings infected by intranasal inoculation, no death occurred in the group, but virus shedding and growth depress was observed (Fig. S4 & S5). These results indicated that the isolate might infect goslings via oral and nasal routes, further demonstrating the infectivity of the isolate.

## Discussion

Several studies have reported the existence of AstV in goose flocks^18, 19^, but the prevalence and pathogenicity of AstV among domestic geese remains poorly understood due to the lack of efficient in vitro culture techniques and diagnostic assays. In this study, AAstV SD01 was successfully isolated by inoculating tissue samples into goose embryos, as confirmed by RNA detection methods similar to those for pan-AstV. We successfully cultured the virus in vitro provided a convenient method for virus propagation and laboratory diagnosis. Isolation of the AAstV from field cases and reproduction of the disease in a gosling infection model with the inoculating virus prepared in embryos fulfils Koch′s postulates.

AstV infection occurred within the first days or week of life usually resulted in a worse outcome, as the age-dependent pathogenicity of AAstV has been reported^10^. In this study, the mortality of young goslings caused by subcutaneous or oral inoculation indicated that AAstV SD01 was highly pathogenic. The experiment was fairly a represent of the situation in the field, where susceptible goslings are exposed to AstV soon after they are placed in contaminated houses. Apart from mortality, avian AstV infection can decrease feed intake and alter feed conversion efficiency, leading to growth repression. The decrease in body weight of infected goslings is a major concern as a 29% lighter body weight at 14 dpi has a considerable economic impact.

Histologic examination revealed the presence of a proteinaceous substance in the renal tubules, indicating that AAstV SD01 infection caused increased permeability of the kidney epithelia barrier. Degeneration and necrosis of tubular epithelial cells found in the deceased goslings provided further evidence of kidney function damage. These results could explain the development of visceral urate deposition in infected goslings. Increased epithelium permeability due to AstV infection has been reported in both human and avian species^24, 25^. Extra-intestinal infection with nephritis has been reported in birds infected with chicken astrovirus and avian nephritis virus^16, 26^. Viral RNA was found in all of the tissues sampled from the infected goslings killed on 5 dpi, indicating that the goose AstV has a wide tissue tropism and spread systemically after inoculation. Virus shedding was detected by RT-PCR and persisted in the infected goslings for about 12 days, further indicating that the virus replicated efficiently in vivo.

It is interesting that encephalitis lesions were observed in the deceased goslings (data not shown), along with the detection of AAstV SD01 RNA in the brain tissue (Fig. S2). However, no neurological symptoms were noted in either the field cases or the experimentally infected goslings. The neurologic infection of AAstV SD01 is worthy of further investigation since there are numerous reported cases of AstV-associated encephalitis and meningitis in humans and mammals^9, 27^. Nonetheless, based on the limited number of goslings infected in present study, it is not likely to get accurate evaluation for the virulence of the isolate.

## Conclusion

The present work describes the isolation of the astrovirus AAstV/Goose/CHN/2017/SD01 from tissue samples of goslings dying from a disease characterized by visceral urate deposition. The successful reproduction of the disease by experimental infection demonstrates the etiological role of this AAstV. Based on the genetic analysis of the complete capsid region at amino acid level, the isolate should be assigned as a member within the genotype consisting of TAstV-2 and DAstV-1 strains. The high variability of the genomic sequence to other known astroviruses suggest more detailed antigenic investigations should be performed.

## Materials and methods

### Bacterial culture and molecular detection of virus genomes

For bacteriological diagnosis, liver, and kidney samples from dead goslings were first inoculated onto tryptic soy agar plates (BD Science, MD, USA) containing 2% fetal calf serum, and incubated at 37 °C under an atmosphere with 5% CO_2_ for 48 h. Then the spleen, liver, and kidney tissue were pooled and tested for the presence of goose parvovirus^28^, goose hemorrhagic polyomavirus^29^, AstV^21^, reovirus^30^, and Tembusu virus^31^, respectively.

### Virus isolation in goose embryos

To isolate AstV, the kidney, spleen, and liver samples were homogenized with sterile phosphate buffered saline (PBS, pH 7.4) to a 20% suspension (w/v) and centrifuged at 8000×*g*, at 4 °C for 10 min. The supernatant was filtered using a syringe-driven filter unit with a pore size of 0.2 μm and the filtrate was inoculated into five 9-day-old goose embryos (0.2 ml/egg) via the chorioallantoic membrane route. Embryos were incubated at 37 °C and candled daily. Embryos that died beyond 24 h and those that survived until 5 day after inoculation were chilled to 4 °C overnight. The allantoic fluids were collected for a hemagglutination (HA) activity test performed by a standard method using 1% chicken red blood cells as an indicator, and then were subjected to additional passage in goose embryos.

To determine the infectious titers of the 4^th^ and 9^th^ passage, the virus suspension was 10-fold serially diluted with PBS and inoculated into 9-day-old goose embryos via the chorioallantoic membrane route. The embryos were incubated for 7 days at 37 °C and the mean embryo lethal dose (ELD_50_) of infectious virus was calculated using the Reed–Muench method^32^.

### Astrovirus genome sequencing

To sequence the complete genome of the isolate, total RNA was extracted from the goose embryo allantoic fluids of the fourth passage using a viral RNA kit (Omega, GA, USA) and the cDNA was synthesized using a Reverse Transcription System (Promega, WI, USA) with random primers following the manufacturer’s instructions. Viral genomic fragments were amplified by PCR with primer sets designed against conserved regions of the AstV sequences retrieved from the GenBank database (Table 2). PCR products were purified using Gel Extraction Kit (Omega, GA, USA) and ligated into *pEASY*-Blunt Simple Cloning Vector (TransGen Biotech, Beijing, China). The recombinant vector was transformed into competent *Escherichia coli* Trans-T1(TransGen Biotech, Beijing, China) and transformants containing the PCR amplified fragment were selected by PCR following the manufacturer’s instruction. At least two representative transformants were subjected to bidirectional DNA sequencing using Applied Biosystems ABI3730 (Shanghai Meiji Biological Medicine Technology Co., Ltd. Shanghai, China). The 5′ and 3′ ends of the viral genome were amplified using the 5′/3′ RACE kit (Clontech, CA, USA) following the guidelines of the manufacturer. The initial complete genome was assembled and manually edited using the Software ContigExpress. Based on the initial genome sequence, additional primer pairs were designed (Table S3), and PCR amplicons were sequenced to determine the genome.

**Table 2:** Primers used for RT-PCR amplification and sequencing of goose astrovirus isolate AAstV/Goose/CHN/2017/SD01

Primer name	Sequence (5′ → 3′)	Location in genome	Product size (bp)
GAstV5race	TCGTTTCAGCGAGCATTGTAGCCTCTGT	385–414	414
GAstV F1	AAAACAGCAATTGGCGTTGA	236–255	
GAstV R1	TTTTTCCCTCAACRATRACAAT	1667–1688	1453
GAstV F2	TGTGGTCTACAGCCTTGA	1404–1418	
GAstV R2	TCAACTTGTTCATCCTTTAC	2792–2811	1408
GAstV F3	AGATTGATGAAGCCATTGAG	2604–2624	
GAstV R3	TGCCGACGCTCAGATT	4340–4355	1752
GAstV F4	ACCATCATAAGACACCACAG	4127–4146	
GAstV R4	TCATTTTGTCATTAACGGG	5014–5032	906
GAstV F5	GGGCGGTGGCCCCGCGCG	4835–4852	
GAstV R5	CTTGACCTGGATTCTGCC	6186–6203	1369
GAstV F6	TACTCCCTCGCTTGTGTACA	6102–6121	
GAstV R6	CTCGGCGTGGCCKCSRCTGCTG	6952–6970	869
GAstV3race	TTGGTCAGTGTCAGATTCC	6630–6648	546
GAstVF7	ATTCTTGGCTCGGTTGTC	5239–5256	
GAstVR7	CCTGTGTTGCTCCTTCTC	5710–5727	489

To evaluate the potential adaptive mutation (s) of the virus that might occur during the process of goose embryo passage, we sequenced the complete genome of initial virus using the total RNA extracted from the clinical case tissue homogenate of kidney, spleen, and liver using the method described above. The genome sequence was compared with that of the isolate of fourth passage.

### Phylogenetic analysis

Nucleotide sequences of the virus genome and the deduced amino acids of the ORFs were compared with known AstV ORF sequences retrieved from the GenBank database. Neighbor-joining trees of the complete genome nucleotide sequences, ORF1b and ORF2 amino acid sequences were constructed using MEGA 7.0 software, with bootstrap values calculated from 1000 replicates. The mean amino acid genetic distance (p-dist) of the viral RdRp and capsid were calculated using MEGA 7.0 with a bootstrap test obtained from 100 replicates.

### Experimental infection study

One-day-old goslings (*Anser anser domesticus*) were obtained from a local hatchery. Birds were raised in negative pressured isolators with ad libitum access to feed and water. Three experiments were conducted to investigate the pathogenicity of the isolate. Animal infection experiments were approved by the China Agricultural University Animal Ethics Committee.

#### Gosling experiment 1

The aim of this experiment was to evaluate whether the isolate was pathogenic in goslings. Thirteen 2-day-old goslings were infected by subcutaneous inoculation with 0.5 ml of the virus suspension prepared from the infected goose embryos at the fourth passage (containing approximately 2.5 × 10^4^ ELD_50_). Goslings inoculated with sterile PBS (uninfected control) were kept in a separate isolator. Clinical signs and mortality were recorded for 10 days. Dead birds were necropsied immediately, and tissue samples of the heart, liver, spleen, kidney, lung, thymus, bursa, and brain were collected. A portion of these tissues were fixed in 10% neutral buffered formalin, embedded in paraffin, and 4–5μm sections were cut and stained with hematoxylin and eosin (H&E). Pieces of liver and kidney tissue were frozen and subjected to virus isolation.

#### Gosling experiment 2

This experiment was designed to investigate the in vivo replication of the virus and its impact on the growth of infected birds. Fourteen 5-day-old goslings were infected as in experiment 1. Birds inoculated with sterile PBS were used as the uninfected control. The tissue distribution of the virus was analyzed in three infected goslings 5 dpi. Representative tissues samples were collected and subjected to histopathological examination and virus RNA detection. For virus shedding detection, cloacal swabs were collected from both infected and control birds on 2, 4, 6, 8, 10, 12, and 14 dpi. Swabs were immersed in 1 ml of 1 × Dulbecco’s modified Eagle’s medium (Gibco, NY, USA) and frozen at −75 °C. Simultaneously, individual birds were weighed. The difference between the mean body weights of infected and uninfected control birds was tested by Student’s *t*-test. Significant differences were defined by *P*-values <0.05 (*), <0.01 (**).

#### Gosling experiment 3

To investigate the possible infection route, three groups of 1-day-old goslings were kept at separate isolator. Birds were inoculated orally (*n* = 6) or intranasally (*n* = 6) at 5-day old with the astrovirus isolate at the dose as experiment 1. Five goslings were kept as uninfected control. Mortality were checked daily. Cloacal swabs were collected for virus shedding detection as described in experiment 2.

### RT-PCR detection of viral RNA in goose tissues

Tissue samples were prepared as a 10% suspension (w/v) in PBS and were homogenized using beads in a high-throughput Tissuelyser (Nibo Scientz Biotechnology Co., Ltd., Zhejiang, China). Then, the homogenate was centrifuged at 8000×*g* for 5 min at 4 °C and 150 μl of the supernatant was used for RNA extraction. Total RNA was extracted and converted to cDNA using the kits described above. PCR amplification was conducted using a set of specific primers (GAstVF7/GAstVR7, Table 2), targeting the *ORF2* gene of the isolate. For virus shedding detection, swab samples were vortexed and centrifuged. After centrifugation, 150 μl of the supernatant was processed for RT-PCR detection in the same manner.

## Electronic supplementary material

Table S1

Table S2

Table S3

Figure S1

Figure S2

Figure S3

Figure S4

Figure S5
