# Supplementary material for: Isolation and characterization of an astrovirus causing fatal visceral gout in domestic goslings
Source: Emerg Microbes Infect. 2018 Apr 19;7:71. doi: 10.1038/s41426-018-0074-5 (PMC5908792; doi:10.1038/s41426-018-0074-5)
Supplement: Supplementary file 1 — Table S1 [file 41426_2018_74_MOESM1_ESM.docx]

**Table S1**  Summary of nucleotide differences in the genome of the initial virus and goose embryo-adapted isolate (4^th^ passage)

| Gene | Nucleotide position | Initial virus | 4^th^ passage | Amino acid mutation |
| --- | --- | --- | --- | --- |
| ORF1a | 1884 | C | T | Synonymous mutation |
| ORF1b | 357 | G | A | Synonymous mutation |
| ORF1b | 750 | T | C | Synonymous mutation |
| ORF2 | 692 | G | A | Nonsynonymous mutation (R→Q) |
| 3＇UTR | 154 | A | G | — |
