# Supplementary material for: Isolation and characterization of an astrovirus causing fatal visceral gout in domestic goslings
Source: Emerg Microbes Infect. 2018 Apr 19;7:71. doi: 10.1038/s41426-018-0074-5 (PMC5908792; doi:10.1038/s41426-018-0074-5)
Supplement: Supplementary file 2 — Table S2 [file 41426_2018_74_MOESM2_ESM.docx]

**Table S2** Pairwise genetic p-distance comparisons of selected astroviruses based on the amino acid sequence of capsid protein

| Virus | 1 | 2 | 3 | 4 | 5 | 6 | 7 | 8 | 9 | 10 | 11 | 12 | 13 | 14 | 15 | 16 | 17 | 18 | 19 | 20 | 21 |
| --- | --- | --- | --- | --- | --- | --- | --- | --- | --- | --- | --- | --- | --- | --- | --- | --- | --- | --- | --- | --- | --- |
| 1.GAstV SD01 |  |  |  |  |  |  |  |  |  |  |  |  |  |  |  |  |  |  |  |  |  |
| 2.TAstV-2 | 0.423 |  |  |  |  |  |  |  |  |  |  |  |  |  |  |  |  |  |  |  |  |
| 3.TAstV-3 | 0.431 | 0.162 |  |  |  |  |  |  |  |  |  |  |  |  |  |  |  |  |  |  |  |
| 4.DAstV-1 | 0.435 | 0.293 | 0.276 |  |  |  |  |  |  |  |  |  |  |  |  |  |  |  |  |  |  |
| 5.GAstV FLX | 0.594 | 0.614 | 0.608 | 0.621 |  |  |  |  |  |  |  |  |  |  |  |  |  |  |  |  |  |
| 6.TAstV-1 | 0.612 | 0.609 | 0.612 | 0.630 | 0.574 |  |  |  |  |  |  |  |  |  |  |  |  |  |  |  |  |
| 7.DAstV-2 | 0.623 | 0.589 | 0.591 | 0.596 | 0.474 | 0.595 |  |  |  |  |  |  |  |  |  |  |  |  |  |  |  |
| 8.DAstV-3 | 0.629 | 0.576 | 0.580 | 0.561 | 0.623 | 0.619 | 0.577 |  |  |  |  |  |  |  |  |  |  |  |  |  |  |
| 9.CAstV GA2011 | 0.636 | 0.610 | 0.612 | 0.618 | 0.602 | 0.644 | 0.623 | 0.360 |  |  |  |  |  |  |  |  |  |  |  |  |  |
| 10.CAstV G059 | 0.640 | 0.587 | 0.594 | 0.599 | 0.520 | 0.587 | 0.510 | 0.583 | 0.612 |  |  |  |  |  |  |  |  |  |  |  |  |
| 11.GfAstV | 0.645 | 0.569 | 0.569 | 0.586 | 0.615 | 0.648 | 0.596 | 0.342 | 0.239 | 0.603 |  |  |  |  |  |  |  |  |  |  |  |
| 12.DAstV-4 | 0.652 | 0.625 | 0.628 | 0.608 | 0.601 | 0.622 | 0.598 | 0.528 | 0.541 | 0.591 | 0.520 |  |  |  |  |  |  |  |  |  |  |
| 13.ANV-2 | 0.687 | 0.709 | 0.704 | 0.713 | 0.705 | 0.636 | 0.687 | 0.701 | 0.693 | 0.698 | 0.696 | 0.680 |  |  |  |  |  |  |  |  |  |
| 14.PhAstV | 0.720 | 0.743 | 0.746 | 0.741 | 0.732 | 0.715 | 0.733 | 0.747 | 0.746 | 0.724 | 0.747 | 0.733 | 0.522 |  |  |  |  |  |  |  |  |
| 15.WpAstV | 0.723 | 0.756 | 0.758 | 0.760 | 0.736 | 0.714 | 0.720 | 0.751 | 0.741 | 0.717 | 0.754 | 0.722 | 0.494 | 0.484 |  |  |  |  |  |  |  |
| 16.ANV-1 | 0.725 | 0.756 | 0.751 | 0.757 | 0.734 | 0.697 | 0.739 | 0.737 | 0.737 | 0.742 | 0.746 | 0.735 | 0.400 | 0.545 | 0.524 |  |  |  |  |  |  |
| 17.ClAstV | 0.727 | 0.741 | 0.736 | 0.747 | 0.728 | 0.664 | 0.728 | 0.730 | 0.715 | 0.731 | 0.720 | 0.722 | 0.345 | 0.527 | 0.475 | 0.411 |  |  |  |  |  |
| 18.Pigeon AstV | 0.729 | 0.754 | 0.751 | 0.757 | 0.736 | 0.703 | 0.739 | 0.743 | 0.745 | 0.747 | 0.751 | 0.738 | 0.394 | 0.550 | 0.532 | 0.047 | 0.420 |  |  |  |  |
| 19.NpAstV MPJ1442 | 0.776 | 0.786 | 0.796 | 0.788 | 0.799 | 0.795 | 0.803 | 0.791 | 0.787 | 0.806 | 0.792 | 0.790 | 0.771 | 0.783 | 0.776 | 0.802 | 0.791 | 0.806 |  |  |  |
| 20.NpAstV MPJ1332 | 0.794 | 0.775 | 0.781 | 0.782 | 0.786 | 0.786 | 0.785 | 0.793 | 0.794 | 0.785 | 0.797 | 0.787 | 0.769 | 0.777 | 0.795 | 0.805 | 0.801 | 0.807 | 0.448 |  |  |
| 21.HAstV | 0.838 | 0.825 | 0.833 | 0.838 | 0.818 | 0.839 | 0.823 | 0.818 | 0.808 | 0.820 | 0.814 | 0.810 | 0.819 | 0.811 | 0.821 | 0.836 | 0.835 | 0.833 | 0.815 | 0.814 |  |

Abbreviations: 1.GAstV SD01, AAstV/Goose/CHN/2017/SD01(MF772821); 2.TAstV-2, VA/99/USA ( EU143851 ); 3.TAstV-3, TAstV-3/Ohio/2001 (AY769616 ); 4.DAstV-1, C-NGB/China/200807 ( FJ919227 ); 5.GAstV FLX, FLX/China/201406 ( KY271027 ); 6.TAstV-1, TAstV-1 ( Y15936 ); 7.DAstV-2, SL5/China/201209 ( KF753807 ); 8.DAstV-3, CPH/China/201306 ( KJ020899 ); 9.CAstV GA2011, GA2011/USA/200708 ( JF414802 ); 10.CAstV G059, Poland/G059/201402 ( KT886453 ); 11.GfAstV, CDB-2012 301-6/Italy/2003 ( JQ307838 ); 12.DAstV-4, YP2/China/201112 ( JX624774 ); 13.ANV-2, ANV-2 /USA/2009（HQ188699; 14.PhAstV, Pond Heron AstV KH08-1279/HK/200810 ( JX985649 ); 15.WpAstV, Wood pigeon AstV 06/15660-1/Oslo/200308 ( FR727147 ); 16.ANV-1, ANV-1/Sichuan/2009 ( HM029238 ); 17.ClAstV, Columba livia AstV KG119/HK/200911 ( JX985647 ); 18.Pigeon AstV, Pigeon AstV SH10/Shanghai/201009 ( HQ889774 ); 19.NpAstV MPJ1442, Northern pintail AstV MPJ1442/HK/200912 ( JX985652 ); 20.NpAstV MPJ1332, Northern pintail AstV MPJ1332/HK/200912 ( JX985650 ); 21.HastV, HAstV ( JN887820 );
