# Supplementary material for: Isolation and characterization of an astrovirus causing fatal visceral gout in domestic goslings
Source: Emerg Microbes Infect. 2018 Apr 19;7:71. doi: 10.1038/s41426-018-0074-5 (PMC5908792; doi:10.1038/s41426-018-0074-5)
Supplement: Supplementary file 3 — Table S3 [file 41426_2018_74_MOESM3_ESM.docx]

**Table S3** Primer pairs designed based on the first round of sequencing results.

| **Primer name** | **Sequence (5′→3′)** | **Location in genome** | **Product size (bp)** |
| --- | --- | --- | --- |
| **1F** | AAACAGCGATATGGCGGC | 1-18 |  |
| **1R** | CCTTCAACAACGACAATGG | 1665-1683 | 1683 |
| **2F** | CAGTCCCTGTACAGATTTTA | 1446-1465 |  |
| **2R** | TCAACTTGTTCATCCTTTAC | 2792-2811 | 1366 |
| **3F** | AGATTGATGAAGCCATTGAG | 2604-2623 |  |
| **3R** | CAGCCCGCCGTTCTGTCTGT | 3939-3958 | 1355 |
| **4F** | AGGCTGTATCAGATATTGAT | 3755-3774 |  |
| **4R** | TCATTTTGTCATTAACGGG | 5014-5032 | 1278 |
| **5F** | GGGCGGTGGCCCCGCGCG | 4835-4852 |  |
| **5R** | CTTGACCTGGATTCTGCC | 6186-6203 | 1369 |
| **6F** | ACAACTGGACAAGGTACC | 6028-6045 |  |
| **6R** | TTTGCGGATTTTAAATGC | 7131-7148 | 1121 |
