# Supplementary figures and images for: Isolation and characterization of an astrovirus causing fatal visceral gout in domestic goslings
Source: Emerg Microbes Infect. 2018 Apr 19;7:71. doi: 10.1038/s41426-018-0074-5 (PMC5908792; doi:10.1038/s41426-018-0074-5)

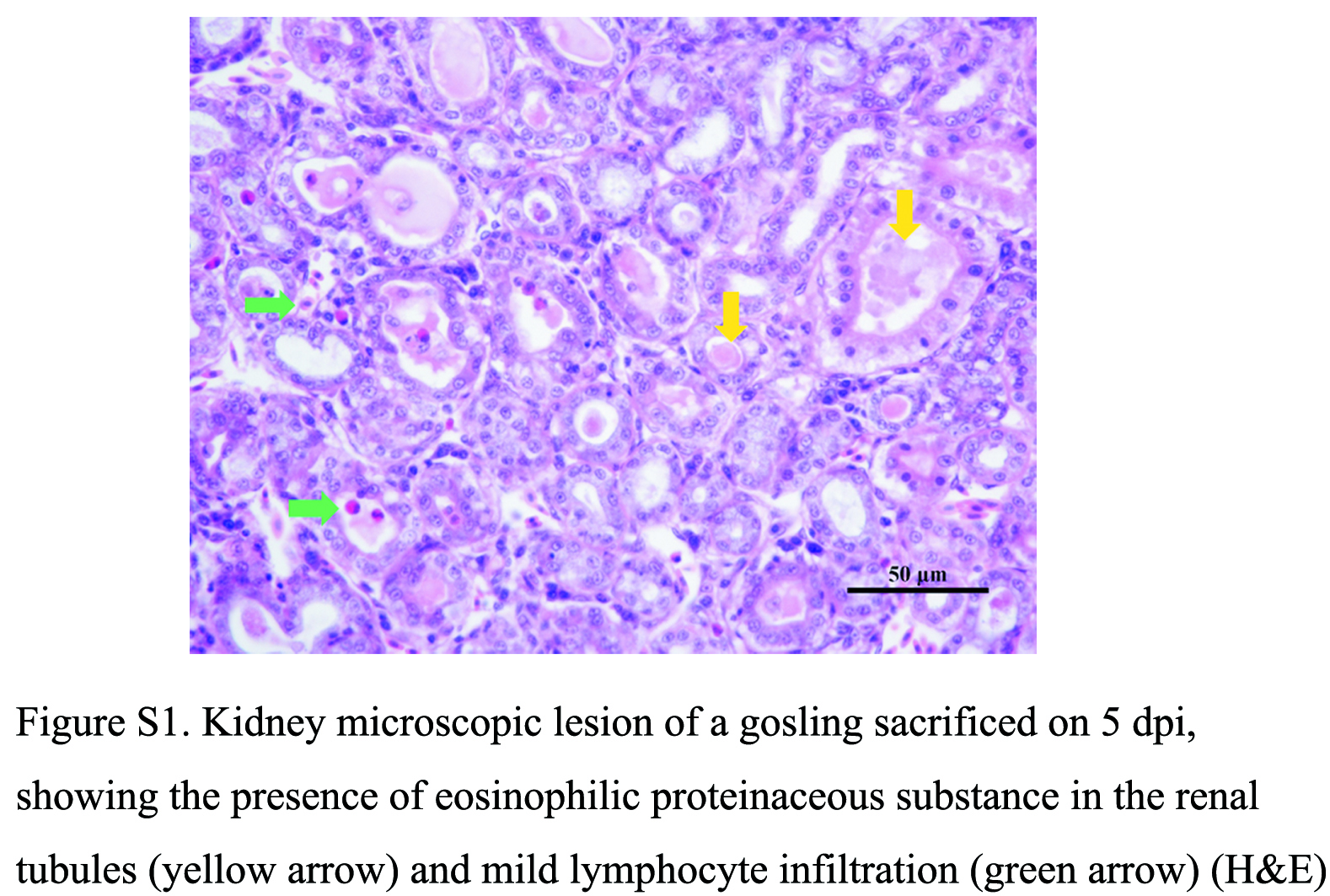

Supplement: Supplementary file 4 — Figure S1 [file 41426_2018_74_MOESM4_ESM.jpg]

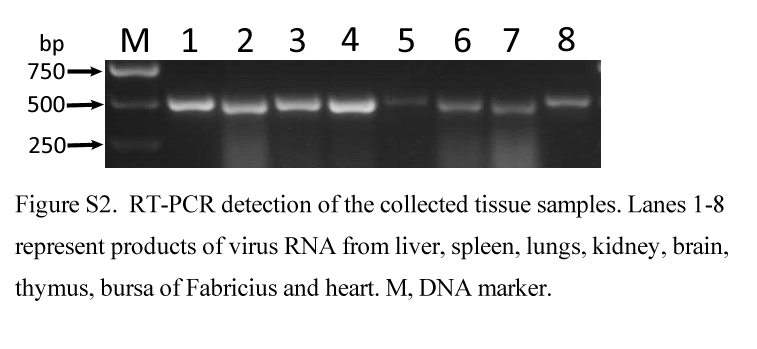

Supplement: Supplementary file 5 — Figure S2 [file 41426_2018_74_MOESM5_ESM.jpg]

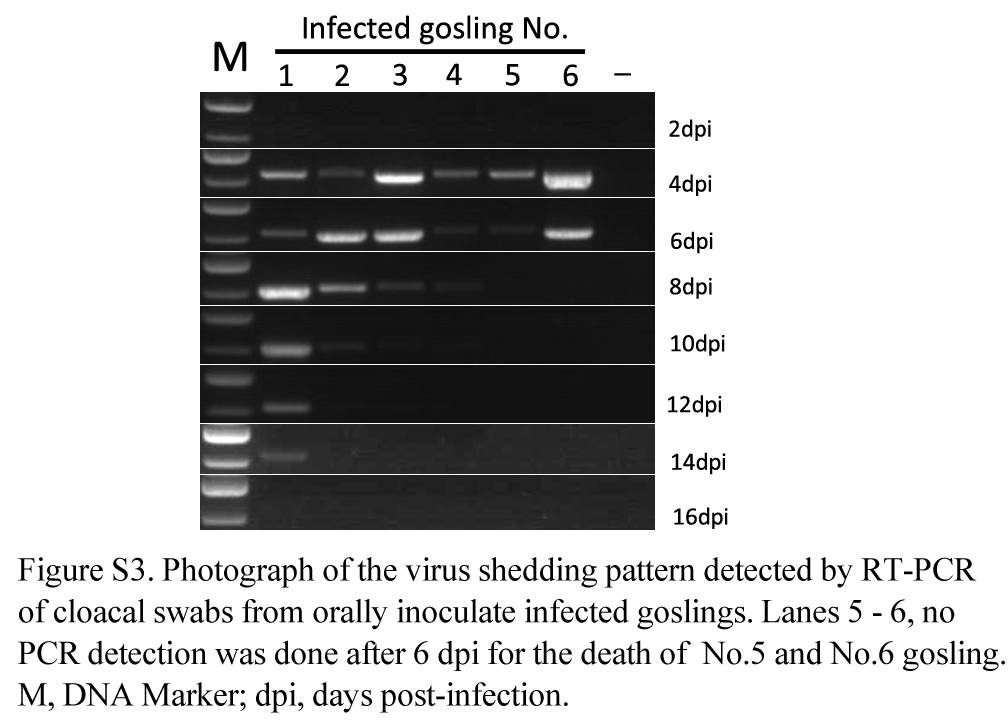

Supplement: Supplementary file 6 — Figure S3 [file 41426_2018_74_MOESM6_ESM.jpg]

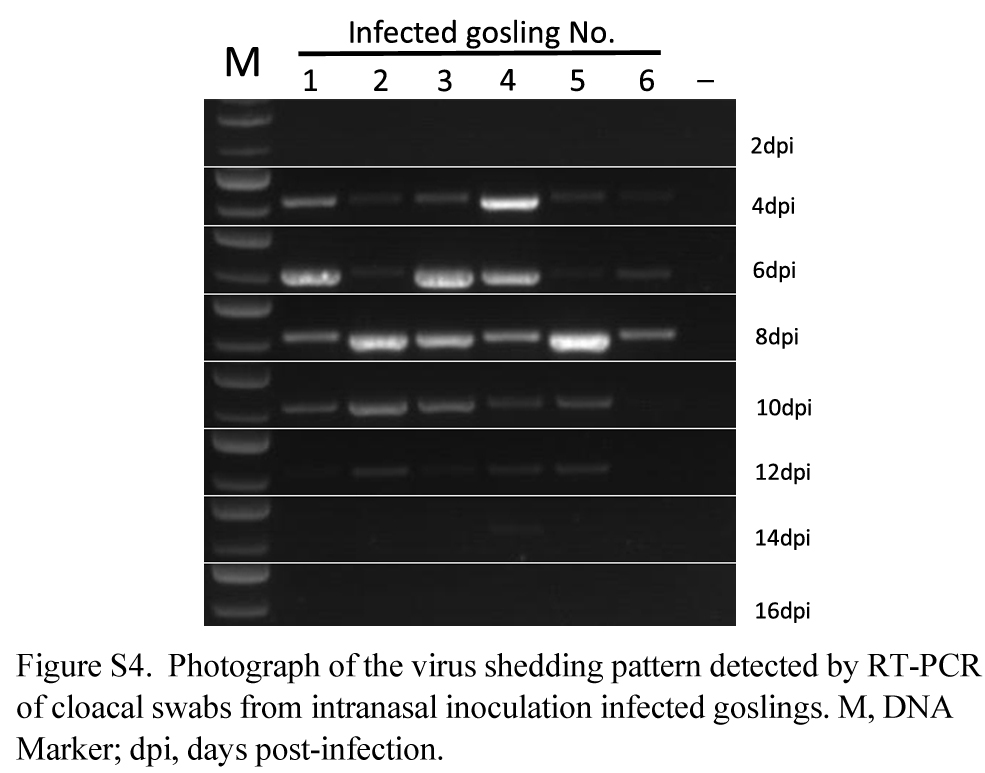

Supplement: Supplementary file 7 — Figure S4 [file 41426_2018_74_MOESM7_ESM.jpg]

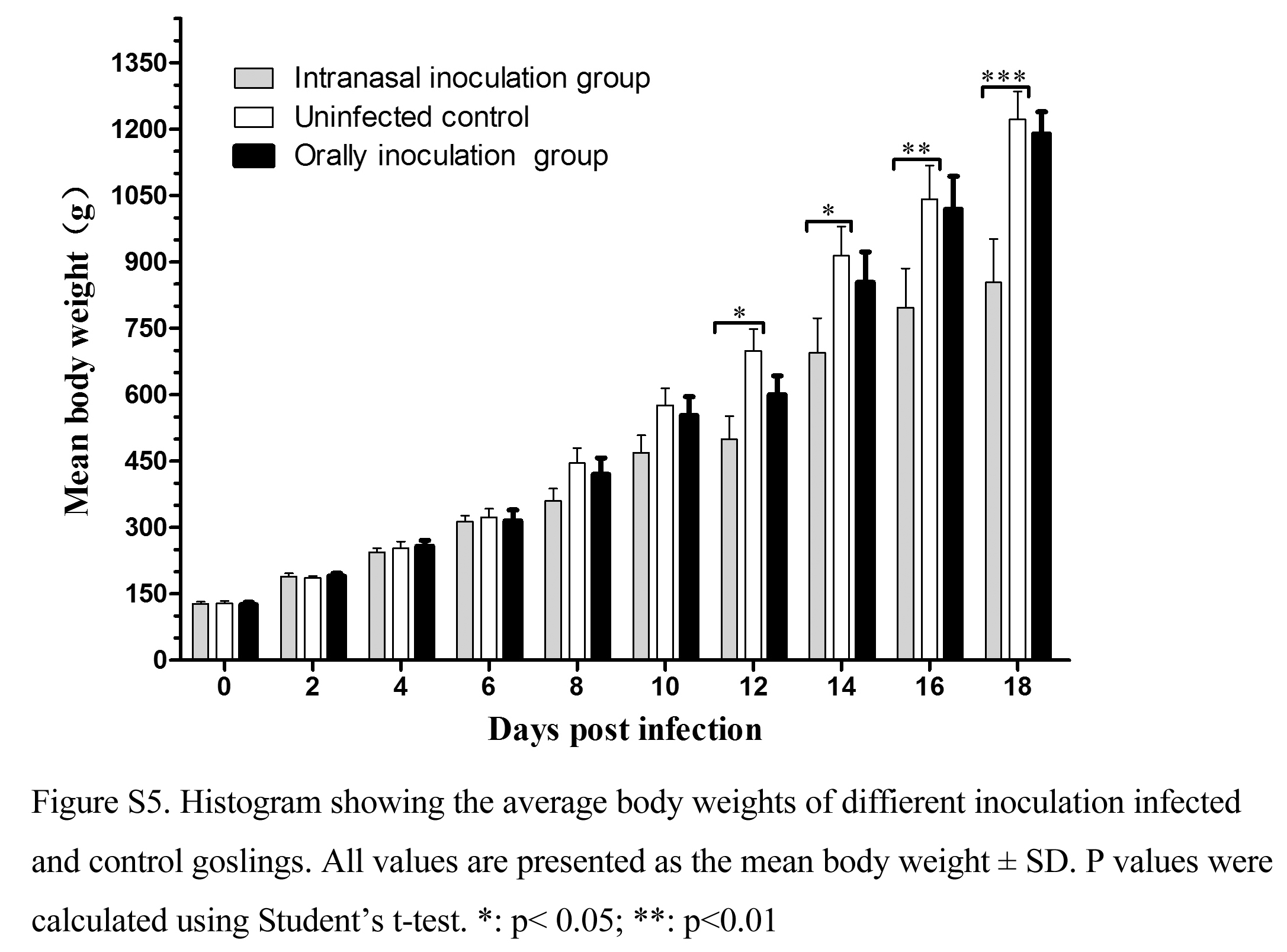

Supplement: Supplementary file 8 — Figure S5 [file 41426_2018_74_MOESM8_ESM.jpg]
